# Presence of 2-hydroxymyristate on endotoxins is associated with death in neonates with *Enterobacter cloacae* complex septic shock

**DOI:** 10.1016/j.isci.2021.102916

**Published:** 2021-07-30

**Authors:** Luis A. Augusto, Nadège Bourgeois-Nicolaos, Aude Breton, Simon Barreault, Enrique Hernandez Alonso, Stuti Gera, Véronique Faraut-Derouin, Nada Semaan, Daniele De Luca, Richard Chaby, Florence Doucet-Populaire, Pierre Tissières

**Affiliations:** 1 Université Paris-Saclay, CEA, CNRS, Institute for Integrative Biology of the Cell (I2BC), 91198, Gif-sur-Yvette, France; 2 Department of Bacteriology-Hygiene, AP-HP Paris-Saclay, Hôpital Antoine Béclère, Clamart Cedex, France; 3 Department of Pediatrics Intensive Care and Neonatal Medicine, AP-HP Université Paris -Saclay, Bicêtre Hospital, Paris, France; 4 Department of Neonatal Intensive Care, AP-HP Université Paris -Saclay, Hôpital Antoine Béclère, Clamart, France; 5 FHU Sepsis, AP-HP/Université Paris-Saclay/Inserm, Le Kremlin-Bicêtre, France

**Keywords:** Molecular Biology, Immunology, Microbiology

## Abstract

*Enterobacter cloacae* complex species are involved in infections among critically ill patients. After a recent *E.cloacae* outbreak of fulminant neonatal septic shock, we conducted a study to determine whether septic shock severity and its lethal consequence are related to structural features of the endotoxin (lipopolysaccharide [LPS]) of the strains isolated from hospitalized infants and more specifically its lipid A region. It appeared that the LPSs are very heterogeneous, carrying fifteen different molecular species of lipid A. The virulence was correlated with a structural feature identified by matrix-assisted laser desorption ionization–time of flight mass spectrometry and gas chromatography coupled with mass spectrometry: the presence of 2-hydroxymyristic acid as a secondary substituent in lipid A. This is the first published evidence linking LPS structural moiety to neonatal sepsis outcome and opens the possibility of using this fatty acid marker as a detection tool for high-risk patients, which could help reduce their mortality.

## Highlights

• Fifteen different molecular species of lipid A is found in *E. cloacae* complex
• 2-Hydroxymyristate moiety on Lipid A is a virulence marker of the *E. cloacae* complex
• Presence of 2-hydroxymyristate is associated with mortality in neonatal sepsis

## Graphical abstract

**Figure:**
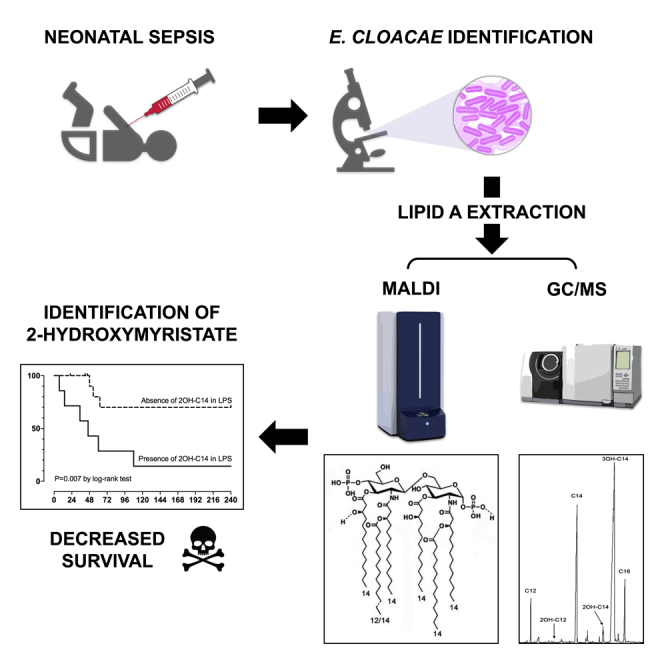

## Introduction

Species of a *Enterobacter cloacae* complex, gram-negative bacteria of the Enterobacteriaceae family, are commonly isolated from soil, plants, and from the digestive tract of mammals and insects [Jang and Nishijima,1990; Marchini et al., 2002]. As a facultative anaerobe, it has the ability to survive in various environments, including dry soil, water pipes, and metal or plastic medical equipment [Herson et al., 1987]. The *E. cloacae complex* is a common catheter contaminant [Watson et al., 2005; Harbarth et al., 1999] and can be an opportunistic pathogen of immunocompromised adults and neonates [Mayhall et al., 1979; Davin-Regli et al., 2019]. The *E.* *cloacae* complex includes various species where *Enterobacter cloacae, Enterobacter bugandensis,* and *Enterobacter hormaechei* represent the most frequently isolated species in clinical infections, especially in the neonatal intensive care unit (NICU) [Davin-Regli et al., 2019; Pati et al., 2018]. In humans, the *E. cloacae* complex is a member of the normal gut microbiota [Keller et al., 1998]. In recent years, the *E. cloacae* complex has emerged as one of the most commonly found nosocomial pathogen in the NICU, but little is known and has been published about its virulence-associated factors [Dalben et al., 2008]. The aim of this study was to search for an eventual correlation between lipopolysaccharides (LPS) structure and the pathogenicity of strains of the *E. cloacae* complex isolated in premature infants with septic shock.

Endotoxins (LPS) are recognized virulence factor by interfering with host recognition, immune response, and action of antimicrobial agents. Modifications of the lipid A region of the LPS molecules are known to modify the penetration ability of some antimicrobial agents [Nikaido, 2003]. Similarly, an increased virulence of Gram-negative bacteria can be due to increased resistance to different host cationic antimicrobial peptides (CAMPs) such as defensins, human neutrophil peptide (HNP-1), human cationic protein 18 (hCAP18 or LL-37), and the human platelet-derived kinocidin [Peschel and Sahl, 2006]. The main mechanisms of resistance to CAMPs consist of LPS modification through the addition of 4-amino-4-deoxy-L-arabinose or phosphoethanolamine, which decreases the negative charge of the lipid A. The operons encoding enzymes involved in these modifications are *arnBCADTEF* and *pmrCAB*, respectively [Olaitan et al., 2014]. Activation of the LPS-modifying genes is often mediated through PmrA/PmrB and PhoP/PhoQ, two-component regulatory systems that are interconnected depending on the species. For instance, in *Escherichia coli*, *Salmonella enterica,* or *Klebsiella pneumoniae*, the phosphorylated form of PhoP can stimulate the expression of PmrD that in turn activates PmrA promoting the transcription of the *arnBCADTEF* and *pmrCAB* operons. Regarding the *E. cloacae* complex and its resistance to CAMPs, it has been shown recently that the *arn* operon is involved, but in contrast to *E. coli*, *Salmonella,* or *Klebsiella*, only PhoP/PhoQ (and not PmrA/PmrB) seems to play a role [Gibbons et al., 2000].

An *E. cloacae* complex outbreak occurred in the NICU of Antoine Béclère Hospital (Clamart, France). The “fulminating” course of nosocomial infection due to the *E. cloacae* complex in critically ill patients urges to investigate the role of endotoxins in the pathophysiology of those infections. Suggested by the fulminating and devastating course of septic shock, we hypothesized that LPSs structures of the patient's isolated *E. cloacae* complex strains, particularly their lipid A moiety, might display some specific structural signatures associated with this severe pathophysiologic response.

## Results

### *Enterobacter cloacae* complex fulminant neonatal septic shock

From January 2016 to June 2017, 18 patients admitted in the NICU of the Antoine Béclère Hospital (AP-HP Paris-Saclay University, Clamart, France) had *E. cloacae* complex septic shock. Patient characteristics are displayed in Table S1. All patients were extremely premature infants (median 27.0 weeks of gestational age). Of the 18 infants, 12 infants displayed a fulminant course with death occurring within a median of 61 h (IQR 22–1062) after septic shock diagnosis. Death occurred on a median of 7 days (IQR 4–10) after birth. Nineteen *E. cloacae* complex strains were isolated from blood cultures. For one patient, we isolated two *E. cloacae* complex strains with different antibiotic-resistant pattern (H7i and H7o). Among the 19 *E. cloacae* complex isolates, we identified *E. hormaechei* (n = 11), *E. bugandensis* (n = 7), and *E. cloacae* (n = 1) (Table S2). We identified 9 enterobacterial repetitive intergenic concensus (ERIC)–polymerase chain reaction (PCR) profiles (Figure S1). In *E. hormaechi* species, 5 profiles were identified with a majority of the profile A (7 of 11, 63.7%). In *E. bugandensis* species, 4 profiles were identified with a majority of the profile E (4 of 7, 57.1%).

Among all *E. cloacae* complex strains, we detected an overproduction of AmpC cephalosporinase contributes to the resistance to third-generation cephalosporins in 8 of 19 strains (42.1%). Phenotypic antibiotic resistance patterns were not evenly distributed among *E. cloacae* complex species. The species *E. hormaechei* showed a higher resistance to third-generation cephalosporins (6 of 11, 54.5%) compared to other species (2 of 8, 25.0%) (Table S2).

### Composition of the molecular species present in the lipid A regions

**Figure 1:**
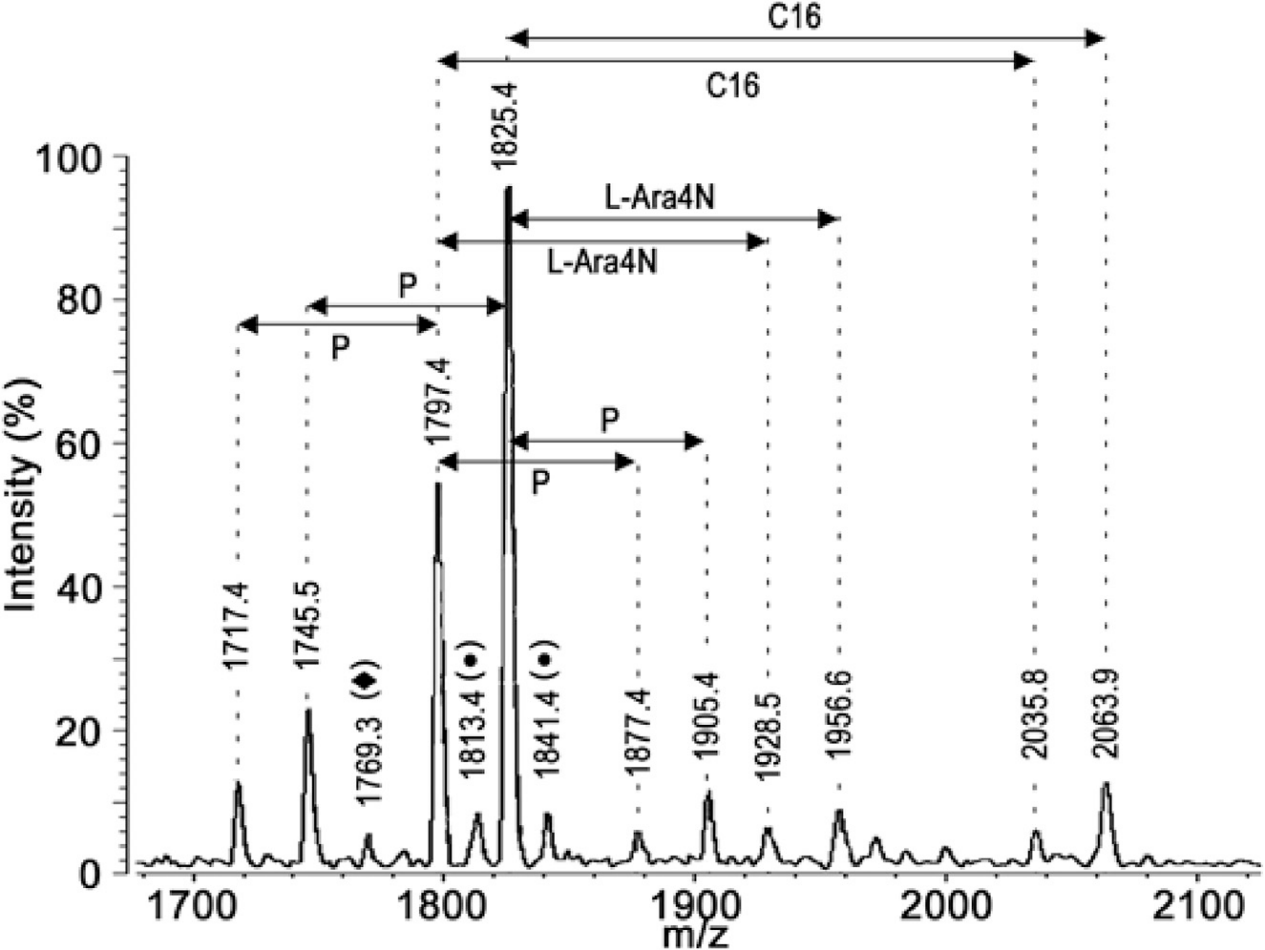
Negative-ion MALDI/TOF mass spectrometry of lipid A from the *E. cloacae* complex H7i (*E. bugandensis* ERIC-PCR Profile low level of chromosomal AmpC beta-lactamase) P, L-Ara4N and C16 represent *m/z* shifts corresponding to phosphate, 4-amino-4-deoxy-L-arabinose and palmitate substituents, respectively. Marked peaks represent species where C14:0 is replaced by C12:0 (⧫) or 2-hydroxymyristate (●).

LPSs were extracted from the cultured strains and the structures of their lipid A regions were analyzed by matrix-assisted laser desorption ionization–time of flioght (MALDI-TOF). Like lipid A of other Gram-negative bacteria, the lipid A moieties isolated from the twelve selected strains of the *E. cloacae* complex contained multiple molecular variants represented by multiple peaks in their mass spectra. Among the twelve lipid A, one of the more heterogeneous was that isolated from strain H7i (*E. bugandensis* E profile with low level of a chromosomal *AmpC* beta-lactamase), with more than 13 significant peaks (13 molecular species) in its lipid A spectrum (Figure 1). The spectrum contained a series of peaks (1360–1388, 1570–1598, 1797–1825, 1928–1956, 2035–2063) with an interpeak distance of 28 mu, suggesting that fatty acids of length differing by two carbons were present in molecular variants of this lipid A. The composition of the molecular species corresponding to the different peaks is indicated in Table 1. The base peak was at *m/z* = 1825 and was likely due to a bisphosphorylated glucosamine disaccharide backbone substituted with two myristic and four hydroxymyristic fatty acids (identified as 3OH-C14 by gas chromatography coupled with mass spectrometry [GC-MS]). This meant that in the homologous peak of the corresponding doublet, at *m/z* = 1797, one of the two myristic (C14:0) acids was replaced with a lauric (C12:0) fatty acid. The following doublets (1928–1956 and 2035–2063) could be easily explained by the addition of palmitate (C16:0) and 4-amino-4-deoxy-L-arabinose (L-Ara4N), respectively, to the *m/z* 1797 and 1825 molecular species.

**Table 1:** Composition of molecular species detectable by MALDI-TOF in Lipid A of *E. cloacae* complex

Constituents	Peaks (calculated m/z)																	
	1388.7	1599.1	1717.4	1745.5	1769.3	1783.4	1797.4	1811.4	1813.4	1825.4	1841.4	1877.4	1905.4	1928.5	1956.6	2035.8	2063.9	2143.8
Total fatty acids	4	5	6													7		
C12			1		2	1	1		1			1		1		1		
C13						1		1										
C14	1	2	1	2			1	1		2	1	1	2	1	2	1	2	2
C16																1	1	1
3OH-C14	3	3	4	4	4	4	4	4	4	4	4	4	4	4	4	4	4	4
2OH-C14									1		1							
Phosphate	2	2	1	1	2	2	2	2	2	2	2	3	3	2	2	2	2	3
L-Ara4N														1	1			

The figures shown represent the number of each residue present in the molecular species characterized by a particular mass peak.

**Figure 2:**
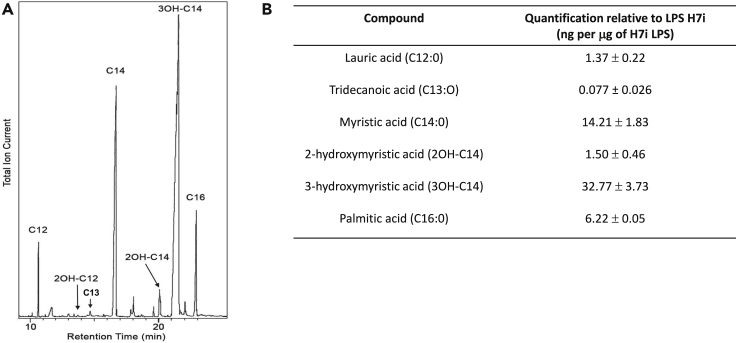
GC/MS analysis of *E. cloacae* complex H7i (*E. bugandensis* ERIC-PCR Profile low level of chromosomal AmpC beta-lactamase) lipid A. (A) Gas chromatogram of fatty acid methyl esters from the H7i LPS of *E. cloacae* complex. C12, C14, 2OH-C14, 3OH-C14 and C16 represent lauric, myristic, 2-hydroxymyristic, 3-hydroxymyristic and hexadecanoic (palmitic) acid respectively. Trace amounts of 2-hydroxylauric acid (2OH-C12) were also detected by the mass spectrometer coupled to the gas chromatograph. (B) Gas chromatogram quantification. Quantification was performed with an internal control based on arachidic (ecosanoic) acid (C20). Values in triplicate are expressed as (mean ± SD).

**Figure 3:**
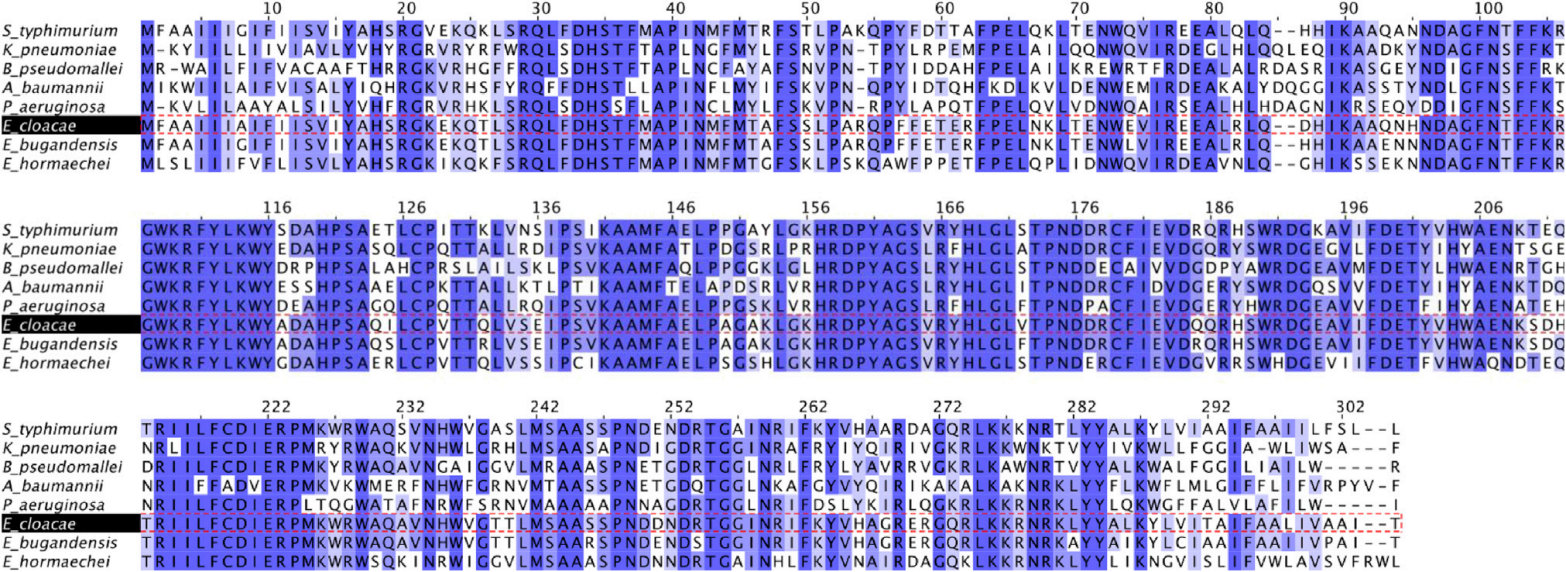
*E. cloacae* complex LpxO homologs in Gram negative bacteria *In silico* analysis revealed that *E. cloacae* complex carries one homolog of known *lpxO* in others Gram-negative bacteria. LpxO homology between *E. cloacae* (GenBank: NC_014121), *S. enterica serovar typhimurium* (GenBank: NZ_CP043907), *K. pneumoniae* (GenBank: NZ_FO834906), *B. pseudomallei* (GenBank: NC_017832), *A. baumannii* (GenBank: NZ_CP059041), *P. aeruginosa* (GenBank: NZ_CP017149), *E. bugandensis* (GenBank: NZ_POUP01000001), and *E. hormaechei* (GenBank: NZ_NJCO01000003).

**Figure 4:**
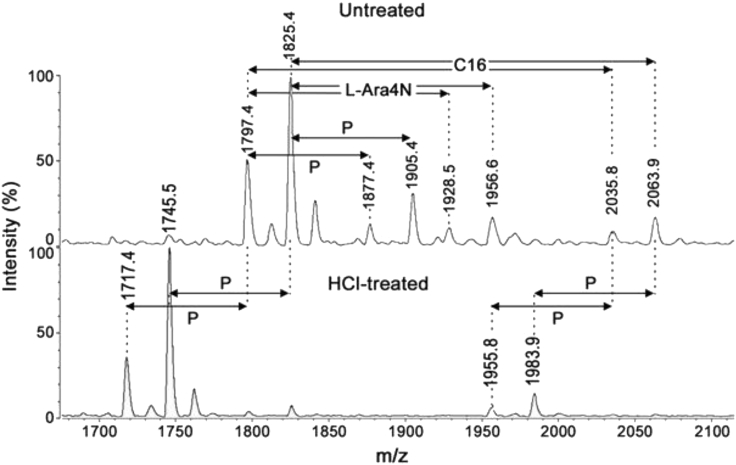
Negative-ion MALDI/TOF mass spectrometry of *E. cloacae* complex H7i (*E. bugandensis* ERIC-PCR Profile low level of chromosomal AmpC beta-lactamase) lipid A, untreated or hydrolyzed with HCl for 10 min at 100°C P, L-Ara4N and C16 represent *m/z* shifts corresponding to phosphate, 4-amino-4-deoxy-L-arabinose and palmitate substituents, respectively.

In addition to the two dominant peaks of the spectrum (at *m/z* 1797.4 and 1825.4), small flanking peaks at +16 Da (*m/z* 1813.4 and 1841.4, marked • in Figure 1) indicating the presence of an alternative hydroxymyristate residue which was used at low frequency in place of C14:0 by a variant acyltransferase. The analysis by GC-MS (Figure 2) indicated the presence in this LPS of trace amounts of α-hydroxymyristic acid (2OH-C14), thus suggesting that a species of lipid A contained this fatty acid and accounted for the small flanking peaks observed. This can be produced by an ortholog of the dioxygenase LpxO identified in *Salmonella enterica* serovar Typhimurium [Gibbons et al., 2000]. *In silico* analyses of several *E. cloacae* complex genomes revealed that this pathogen carries one homolog of known *lpxO* in others Gram-negative bacteria. Blast analysis of *E. cloacae* strain ATCC 13047 showed that LpxO (GenBank: NC_014121) is 84%, 63%, 57%, 56%, and 57% identical to *S. enterica* serovar Typhimurium, *K. pneumoniae*, *Burkholderia pseudomallei*, *Acinetobacter* *baumannii,* and *Pseudomonas aeruginosa* LpxO, respectively (Figure 3). Among *E. cloacae* complex species, *E. cloacae* LpxO shared 90% and only 67% sequence identity with *E. bugandensis* (GenBank: NZ_POUP01000001) and *E. hormaechei* LpxO (GenBank: NZ_NJCO01000003), respectively. LpxO is known to generate 2-hydroxymyristate by hydroxylation of the myristate transferred to lipid A by the acyltransferase MsB/LpxM17. Another small peak (*m/z* 1769.3) in this spectrum (marked ⧫ in Figure 1) can be explained by the presence of a minor species containing two secondary C12:0 instead of the C12 + C14 (*m/z* 1797.4) or C14 + C14 (*m/z* 1825.4) present in the major species. Position of the aminoarabinose and phosphate residues of the lipids A. Monophosphorylated species of lipid A can be produced by acid hydrolysis (0.1 M HCl for 10 min at 100°C). Labile linkages such as pyrophosphates and the acetal linkage of the proximal glucosamine (phosphate linked to C1) are hydrolyzed under these conditions. After such a treatment of *E. cloacae* complex lipid A, the peaks corresponding to the bisphosphorylated and hexa-acylated species containing aminoarabinose (*m/z* 1928.5 and 1956.5) were completely absent from the MALTI-TOF spectrum (Figure 4). Therefore, the L-Ara4N group was not located on P4′ in the untreated bisphosphorylated species because the L-Ara4N→phosphate and the phosphate→4′-GlcN linkages are both resistant to this moderate acid hydrolysis. The loss of phosphoryl-aminoarabinose by mild acid hydrolysis proved that the L-Ara4N substituent was on phosphate at position 1 of the bisphosphorylated species.

Regarding phosphate groups, the presence of triphosphorylated molecules in the untreated lipid A (*m/z* 1877.4 and 1905.4) indicates that a pyrophosphate must be present in these two molecular species. However, we can note that molecules containing pyroposphate (*m/z* 1877.4 and 1905.4) do not contain L-Ara4N, and molecules containing L-Ara4N (*m/z* 1928.5 and 1956.5) do not contain a pyrophosphate. This suggests that during the biosynthesis of the *E. cloacae* complex lipid A, either a third phosphate group or an L-Ara4N group is added on phosphate at position 1. It is noteworthy that the moderate hydrolytic procedure used here did not induce important cleavage of fatty acids since the hepta-acylated and monophosphorylated species (*m/z* 1955.8 and 1983.9) were still present after this hydrolysis.

### Analysis of the acylation patterns of the lipids A

**Figure 5:**
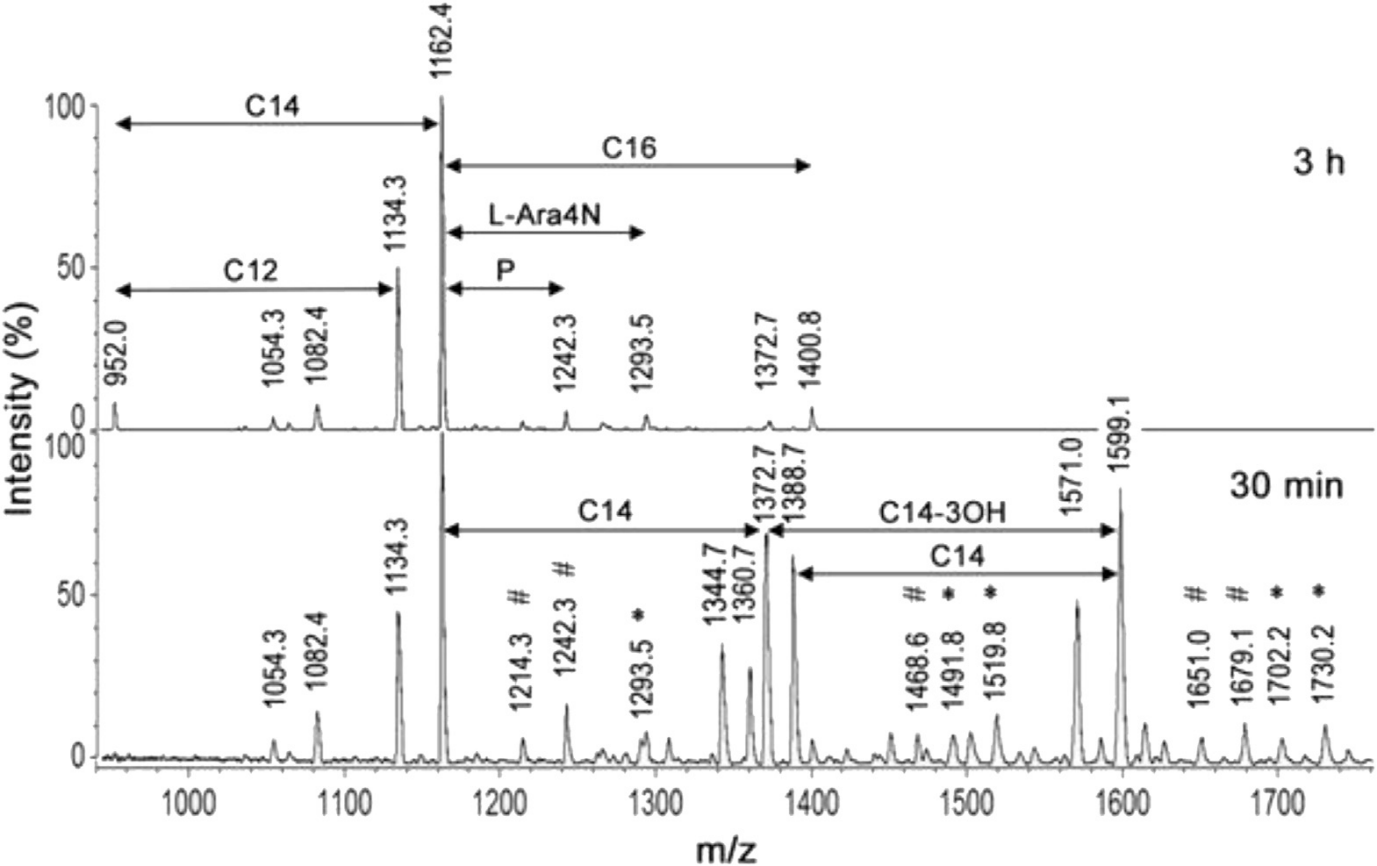
Negative-ion MALDI/TOF mass spectrometry of *E. cloacae* complex H7i (*E. bugandensis* ERIC-PCR Profile low level of chromosomal AmpC beta-lactamase) lipid A treated with ammonium hydroxide (28%, 50°C) for 30 min or 3 h Marked peaks represent molecular species containing two phosphates and one L-Ara4N (∗) or three phosphates without L-Ara4N (#). P, L-Ara4N, C12, C14 and C16 represent *m/z* shifts corresponding to phosphate, 4-amino-4-deoxy-L-arabinose, laurate, myristate and palmitate substituents, respectively.

**Figure 6:**
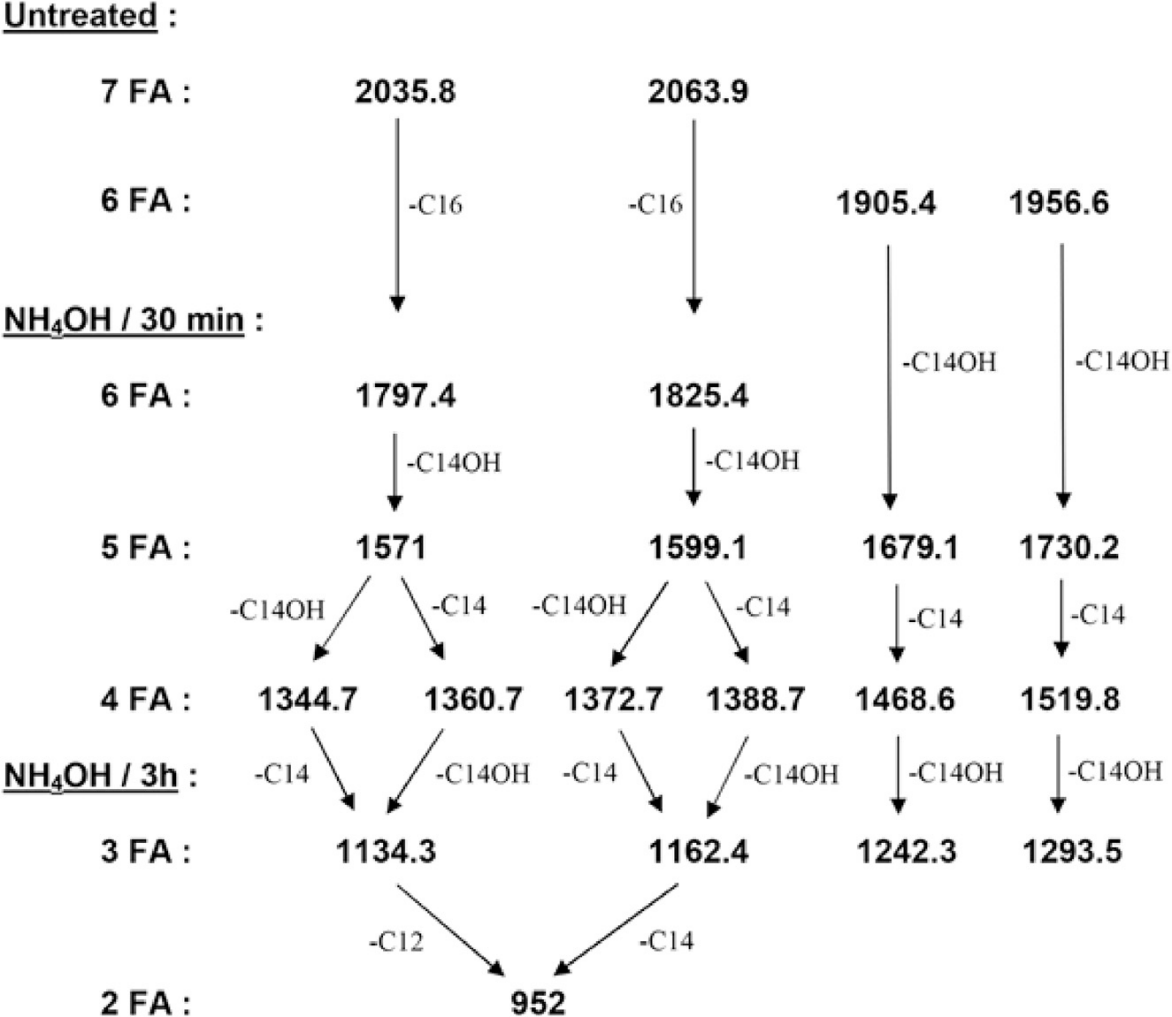
Pattern of cleavage of the molecular species present in the *E. cloacae* complex H7i (*E. bugandensis* ERIC-PCR Profile, with low level of chromosomal AmpC beta-lactamase) during alkaline treatment Numbers are the *m/z* values observed in MALDI-TOF obtained after treatment with 28% NH_4_OH at 50°C for 30 min or 3 hr (see Figure 5). The number of fatty acids (FA) in each species is indicated.

**Figure 7:**
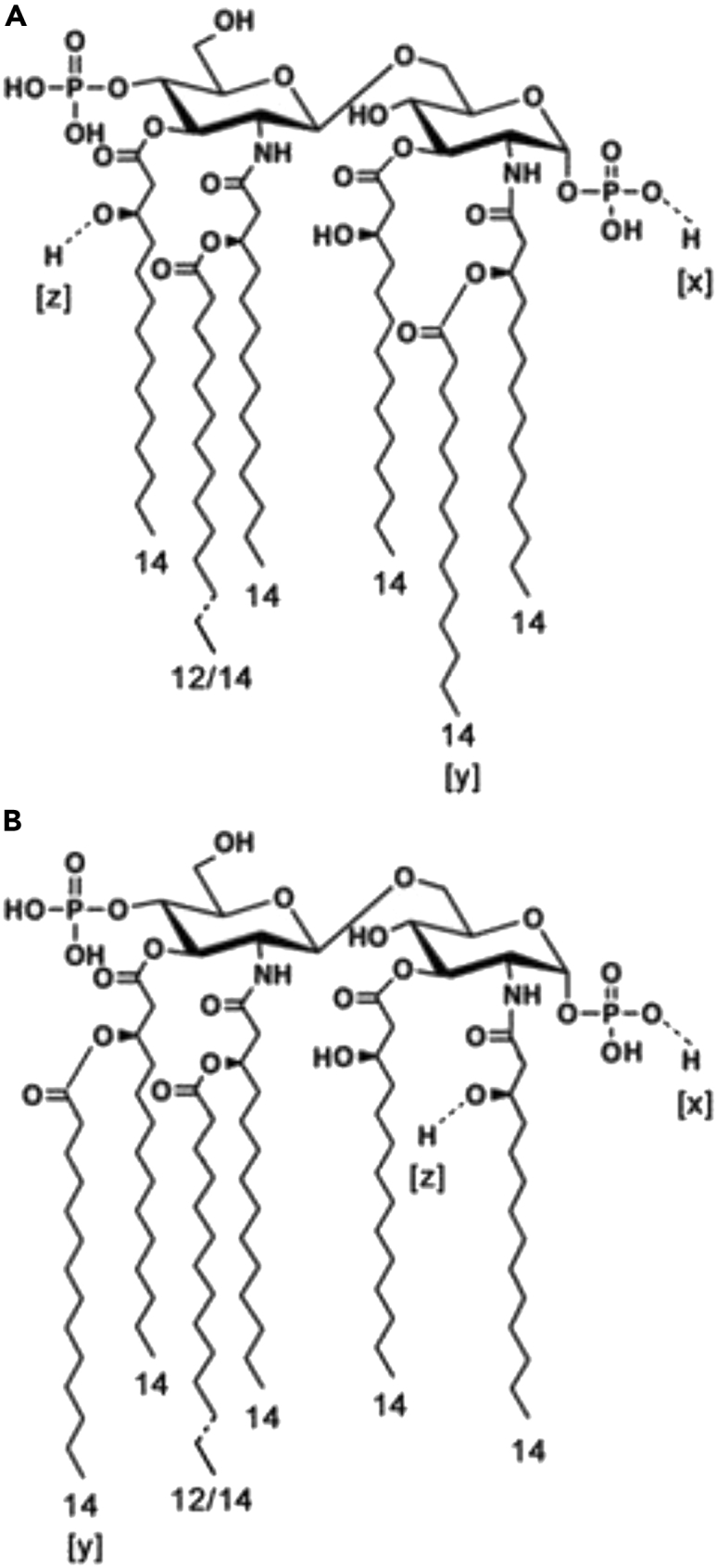
Structure of the major molecular species in the *E. cloacae* complex H7i (*E. bugandensis* ERIC-PCR Profile; with low level of chromosomal AmpC beta-lactamase) Structures (A and B) are present in almost equal amounts. [x] minor substituents: L-Ara4N/PO3H2; [y] minor variants: C12:0/C13:0/2OH-C14; [z] minor substituent: C16:0.

The sequential liberation of ester-linked fatty acids by mild alkaline treatment, as used in previous studies [Olaitan et al., 2014; Silipo et al., 2002], usually provides valuable information on the positions of the different fatty acids on the lipid A backbone. According to these studies, the more resistant are the secondary fatty acids on C2′ and C2 (particularly C2′), and the more labile are those on C3 (either primary or secondary), substitutions on C3′ showing intermediate behaviors. In addition, secondary fatty acids are more resistant to alkaline treatments than primary fatty acids. In order to analyze the acylation patterns of the fatty acids in *E. cloacae* complex LPS, we treated the H7i lipid A with 28% NH4OH at 50°C for 30 min or 3 h (Figure 5 and Figure 6). When the treatment was performed for only 30 min, new hexa-, penta-, and tetra-acylated species were produced. Important information on this lipid A structure and composition can already be gained from the group of four peaks of *m/z* between 1340 and 1390. The peak at *m/z* 1344.7 (two phosphates, two hydroxymyristates, one C12:0, and one C14:0) indicate that only C12:0 and C14:0 are the secondary substituents at 2 and 2′ of the two remaining, amide-linked, hydroxymyristate. The lauric residue C12:0 must be on C2′ because this position is the most resistant to alkaline hydrolysis and C12:0 remains present in several other de-O-acylated fragments (peaks at *m/z* 1797.4, 1571 and 1360.7), even after 3 h of hydrolysis (peak at *m/z* 1134.3). One myristic residue must also be present on the same 2′ position because it is also found after a 3-h hydrolysis (triacylated species at *m/z* 1162.4 with only one C14:0). Because the 2′ position is substituted by either C12:0 or C14:0, it follows that in the species at *m/z* 1344.7 mentioned above, the C14:0 residue is at position 2. The peak at 1372.7 represents the homologous species with secondary C14:0 on 2 and 2'. Therefore, two major molecular species of this lipid A contain a C14:0 on position 2. On the other hand, the peak at *m/z* 1360.7 (two phosphates, three hydroxymyristates and one C12:0) results from the easy loss of only one hydroxymyristate at position 3 (peak at *m/z* 1571.0) followed by the cleavage of a C14:0. Because secondary substituents at position 2 are firmly linked, the cleaved myristate was not initially at position 2 but on the only remaining position, 3'. Therefore, the peak at *m/z* 1360.7 containing C12:0, and its homolog at *m/z* 1388.7 containing C14:0, derive from two other major molecular species, different from those mentioned in the preceding paragraph, which contain a C14:0 on position 3′ and an unsubstituted primary hydroxymyristate on position 2. This means that the group of four peaks of *m/z* between 1340 and 1390 represent four major and structurally different molecular species present in this lipid A. In two of those (general structure A), a secondary C14:0 is at position 2, whereas in the two others (general structure B), it is at position 3' (Figure 7).

Regarding the addition of an acyl-oxo-acyl C14 at the 2 position, the observed peak at m/z 1344.7 (Figure 5) found after mild alkaline treatment (30 min NH4OH) of *E. cloacae* complex lipid A can only result from structure A (shown in Figure 7) after the loss of the labile substituents on C3 and C3' (see scheme in Figure 6). As mentioned above, m/z 1344.7 correspond to a molecule with a C14:0 residue linked to the C14OH at position 2 (it is not located at position 2' and would have been lost during this treatment if located elsewhere). Therefore, between the two main structures of *E. cloacae* lipid A, one is palmitoylated (Figure 7B) and the other is myristoylated (Figure 7A) on the C14OH at C2. In the latter case, the addition of a C14 acyl-oxy-acyl at the C2 position is rare but not novel. A similar feature has been described in *Vibrio cholerae* O 1 lipid A, due to the activity of MsbB [Matson et al., 2010; Tanamoto et al., 2001].

Regarding the palmitic acid present in the hepta-acylated species of lipid A (peaks at *m/z* 2035.8 and 2063.9), after treatment with NH_4_OH for 3 h, we observe small peaks at 1372.7 and 1400.8 representing bisphosphorylated and diacylated molecules containing one C12/C14 and one palmitate (C16:0). The fact that C16:0 remains after this harsh treatment indicate that this palmitic group is on position 2. This can only occur in the general structure B because position 2 is already occupied by C14:0 in structure A. Concerning the latter, two positions are available to accommodate C16: 3 and 3'. After 30 min of NH_4_OH treatment, small peaks at *m/z* 1809.4 and 1837.5 are visible (not shown) and are attributable to hexa-acylated species containing a secondary C16:0 at position 3' (because the hydroxymyristic acid at position 3 is the absent). In conclusion, when present as a minor substituent in lipid A, C16:0 is on 3′ in structure A and on 2 in structure B.

After the cleavage of ester-linked fatty acids with NH_4_OH for 30 min (Figure 6), a series of five small peaks attributable to bisphosphorylated molecules with one L-Ara4N (*m/z* 1730.2, 1702.2, 1519.8, 1491.8, and 1293.5) and to trisphosphorylated molecules without L-Ara4N (*m/z* 1679.1, 1651.0, 1468.6, 1242.3, and 1214.3) were visible in the spectrum (marked with ∗ and #, respectively, in Figure 4). In contrast, species with three phosphates and one L-Ara4N were not detected. This is in line with the absence of such molecules in the untreated lipid A (Figure 1) and strongly suggests that the phosphate at position 1 can be substituted either by an L-Ara4N group, or by a phosphate group, but not by a phosphoryl aminoarabinose.

After this step of our analysis, the complete structures of the main molecular species present in *E. cloacae* complex lipid A can now be proposed (Figure 7). Four major and structurally different molecular species are present: In two of these (general structure A), a secondary C14:0 is at position 2, whereas in the two others (general structure B), it is at position 3'. In addition, when present as a minor substituent, a palmitic group (C16:0) is on position 3′ in structure A and on position 2 in structure B.

### Similarities and differences in lipid A spectra from *E. cloacae* complex selected strains

Comparison of the lipid A spectrum of the twelve *E. cloacae* complex selected strains in this first step of our study indicated that some peaks were not always present, and additional small peaks were sometimes detectable (Table 2). A tetra-acylated (three hydroxymyristic and one myristic fatty acids) and bisphosphorylated glucosamine disaccharide (peak at *m/z* = 1388.7) is present in five strains (C17, C12, H8, H7o, and H10) but absent from the seven others. It should be noted that in *E. coli*, a tetra-acylated and bisphosphorylated GlcN-disaccharide (precursor IVA) plays an important role in the biosynthesis of the core region (attachment of two Kdo residues to precursor IVA). However, the tetra-acylated precursor IVA of *E. coli* contains four 3OH-C14, whereas the tetra-acylated molecular species observed in these five strains of *E. cloacae* complex contains three 3OH-C14 and one C14:0. The formation of this tetra-acylated species is most likely due to the loss, by enzymatic cleavage, of a myristoxy-myristoyl residue from the hexa-acylated form of lipid A at *m/z* 1825.4. Such a cleavage requires the general structure B displayed in Figure 7, which carries a secondary C14:0 on 3'. No correlation was found between this enzymatic cleavage detected in only five strains (C12,C17, H7o, H8, and H10) and the ERIC-PCR profiles of these strains (profiles H, E, E,C, and F, respectively) and the identification *E. cloacae* complex species.

**Table 2:** Peaks present in MALDI-TOF spectra of lipid A isolated from various *E. cloacae* complex strains

	Source of isolation											
	Cavum				Blood							
Strain designation	C17	C18	C16	C12	H1	H2	H11	H9	H8	H7o	H7i	H10
Identification species^a^	NP	NP	NP	NP	E.h	E.h	E. h	E.b	E.h	E.b	E.b	E.b
ERIC-PCR profile	E	F	G	H	A	A	A	B	C	E	E	F
Overproduction of AmpC^a^	N	N	N	N	Y	Y	Y	N	N	Y	N	N

*m/z* (calculated)	Peaks present in the spectra											
1388.7	+			+					+	+		+
1599.1												+
1717.4	+		+	+							+	
1745.5	+	+	+	+		+			+	+	+	+
1769.3	+	+		+					+	+	+	
1783.4				+								
1797.4	+	+	+	+	+	+	+	+	+	+	+	+
1811.4				+		+			+			
1813.4										+	+	+
1825.4	+	+	+	+	+	+	+	+	+	+	+	+
1841.4					+					+	+	+
1877.4		+		+				+	+	+	+	+
1905.4		+	+	+	+	+		+	+	+	+	+
1928.5		+			+	+	+	+	+	+	+	+
1956.6		+			+	+	+	+		+	+	+
2035.8	+	+		+		+		+	+	+	+	+
2063.9	+	+	+	+	+	+	+	+	+	+	+	+
2143.8		+							+			+

a N: No; Y: Yes. NP, not performed; E.h. *Enterobacter hormaechei*; E.b, *Enterobacter bugandensis*.

The presence of other small peaks represents a second type of structural variation between the twelve strains. The small peaks at *m/z* 1813.4 and 1841.4 mentioned above in the spectrum of strain H7i (presence of an α-hydroxy myristic acid), were actually present in four strains: H1, H7o, H7i, and H10 (Table 2). They are due to a 2-hydroxymyristic acid (2OH-C14) which takes the place of the secondary C14:0 in positions 2 (Figure 7A) or 3' (Figure 7B). The same C14:0 can also be replaced by C13:0 (peak at 1811.4 in strains C12, H2 and H8) or by C12:0 (peak at *m/z* 1769.3 in strains C17, C18, C12, H8, H7o, and H7i) (Table 2 and Figure 6).

Analysis of the fatty acid composition of LPS by GC-MS confirmed the results obtained by MALDI-TOF, including the presence of small amounts of tridecanoic and 2-hydroxymyristic groups in some LPSs (Figure 2). Trace amounts of a 2-hydroxylauric group (2OH-C12), undetectable by MALDI-TOF, were also detected by GC-MS in the LPSs of strains C12, H1, H9, H7o, H7i, and H10. Other variations among the twelve isolates are produced by the absence of significant MALDI peaks characteristic of the most complete molecular species. This is the case for the absence of pyrophosphate (trisphosphorylated species at *m/z* 1877.4 and 1905.4) in strains C17 and H11 and also the absence of L-Ara4N (peaks at *m/z* 1928.5 and 1956.6) in strains C17, C16, and C12 (Table 2).

We observed no correlation between the lipid A structures of the different strains, as characterized by their mass spectrum, and their other available features such as their ERIC-PCR profile (A to H), the source from which they were isolated (cavum or blood), or the expression of the cephalosporinase (inducible or overproduced). For example, strains H7o and H7i (isolated from the same infant) have the same ERIC-PCR profile and very similar lipid A mass spectra but differ for the expression of cephalosporinase. Conversely, strains H11 and H7o both have overproduced cephalosporinase but have different lipid A spectra and ERIC-PCR profiles (profiles A and E, respectively).

### Immunological effects of LPSs isolated from the *E. cloacae* complex strains

**Figure 8:**
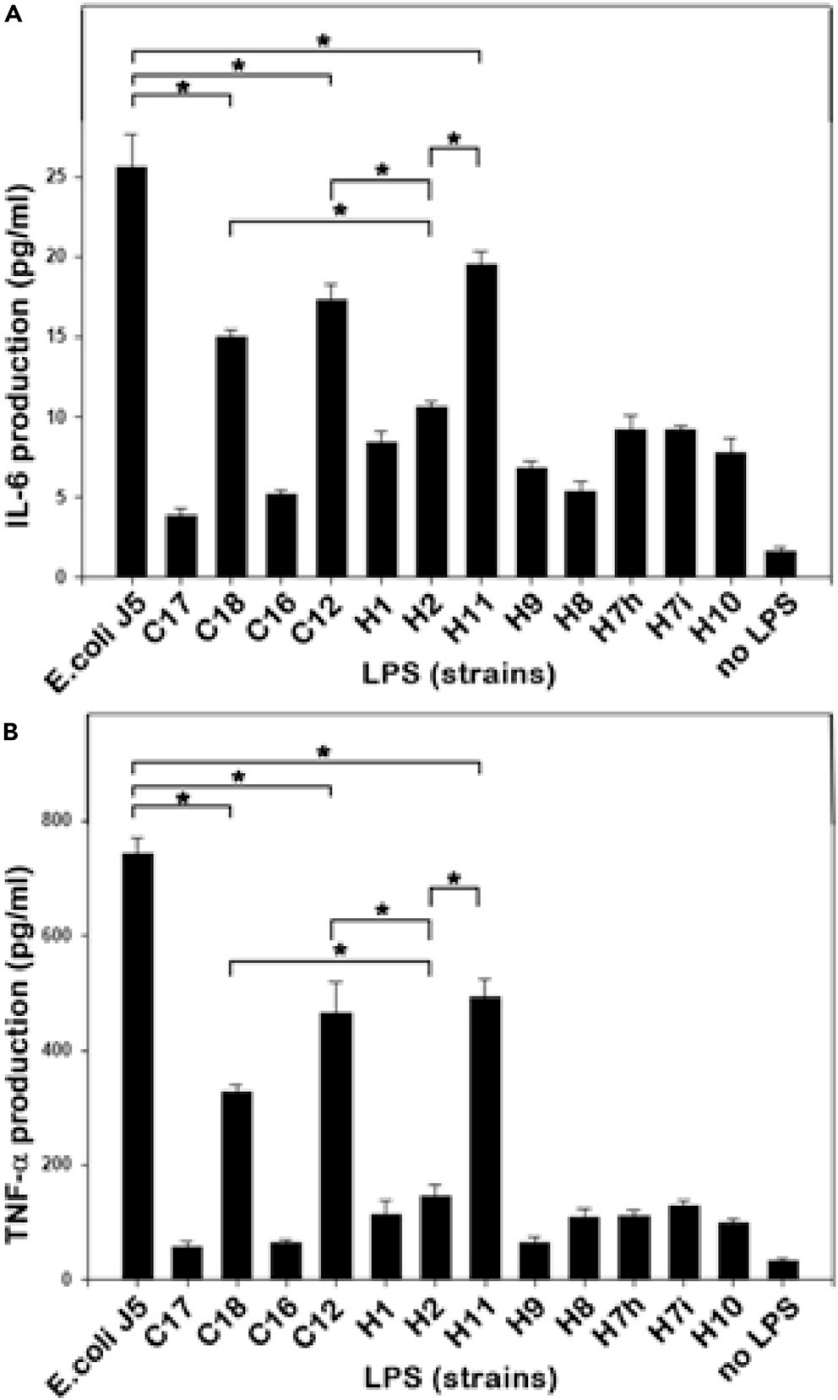
Cytokines secreted by THP-1 cells stimulated by LPSs from various *E. cloacae* complex strains 10^6^ THP-1 cells were stimulated for 24 hr with 1 ng/mL of purified LPS from different *E. cloacae* complex strains or from *E. coli* J5. (A) IL-6 production. (B) TNF-α production. Results are expressed as the mean and standard deviation of 9 values (Three independent experiments in triplicate). Significance was determined using the GraphPad Prism8 software, by two-tailed Student's t-test. ∗: p < 0.0001.

Early biological disturbances in infected newborns (neutropenia, increased C-reactive protein, and procalcitonin), as well as the subsequent observation of a septic shock syndrome, led us to evaluate the production of proinflammatory cytokines (TNF-α and IL-6) induced by LPSs from the infecting strains. The cytokines were measured *in vitro* after stimulation of monocytic THP-1 cells by purified LPSs extracted from the twelve strains of the *E. cloacae* complex. At high concentrations (>20 ng/mL), the twelve LPSs activated the cells and induced TNF-α and IL-6. To differentiate the activities of these LPSs, we analyzed their inflammatory capacity at low stimulation dose: 1 ng/mL (Figure 8). The results show that the relationship between the structure of lipids A and the proinflammatory power of LPS cannot be explained by the presence of a particular molecular species of lipid A or a particular constituent but involves the contribution of all the different molecular species that compose each lipid A. Only three LPSs, extracted from strains C18, C12, and H11, were able to induce significant production of TNF-α and IL-6 (Figure 8), although at a lower level than *E. coli* J5 LPS stimulation. Since each LPSs extract consists of several molecular species, we can consider that the overall inflammatory response (e.g. IL-6 production) is the sum of the contributions of each molecular species. In Figure 8, the high proinflammatory activity of LPSs C18 and H11 can be explained by the presence of the species with *m/z* 1928.5 and 1956.6 (Table 2) with a high contribution factor of 71.5 (Table 3), which both contain an L-Ara4N residue (Table 1). However, the presence of L-Ara4N is neither necessary (see LPS C12) nor sufficient (see for instance LPSs H9 and H10) to trigger a strong proinflammatory response. For example, in the strong inflammatory response secondary to LPS C12 stimulation (Figure 8), the absence of L-Ara4N can be overcome by the presence of other species with high contribution factors such as those with *m/z* 1388.7 and 1717.4.

**Table 3:** Contribution of the different molecular species of lipid A to the overall pro-inflammatory activity of the LPSs of *E. cloacae complex* strains

Molecular species (*m/z*)	Contribution factor to IL-6 production
1388.7	+78.4
1599.1	0
1717.4	+78.4
1745.5	+16.8
1769.3	- 3.6
1783.4	0
1797.4	- 41.2
1811.4	- 16.5
1813.4	- 44.6
1825.4	- 41.2
1841.4	- 44.6
1877.4	- 3.5
1905.4	+33.4
1928.5	+71.5
1956.6	+71.5
2035.8	- 42.7
2063.9	- 41.2
2143.8	- 5.0

### Presence of 2-hydroxymyristate is associated with *E. cloacae* complex neonatal sepsis mortality

Although all the strains selected carry the same major molecular species of lipid A, they differ in minor variants, such as the presence of an L-Ara4N or a pyrophosphate in position 1 and the presence of a 2-hydroxymyristate (2OH-C14) or a C13:0 replacing a myristate (Figure 6). It is therefore possible to classify the strains as a function of the presence in their lipid A of molecular species bearing or not some of these four constituents. By this method, the twelve strains can be clustered into six groups (Table S3). Remarkably, the lethal strains are not distributed randomly in the 6 groups identified but belong to only two groups. This result may suggest that the presence of 2-hydroxy myristate (2OH-C14) (100% [3 of 3] death) and, to a lesser extent, tridecanoate (C13) (1 of 3 death; 33%) markedly enhances the pathological power of the strain as compared to strains without these fatty acids (0% death).

To ensure that there was a correlation between strain lethality and a structural element of its LPS, *E. cloacae* strains isolated from 18 bacteremic infant (septic shock group) and 18 nonbacteriemic infants (colonized group) were analyzed. Nonbacteriemic infants were matched with bacteriemic infants using the Clinical Risk Index for Babies II score in order to have two homogeneous populations for comparisons (Table S1). Strains from the septic shock group were isolated from the blood culture, while in the colonized infants, 9 strains were isolated from the cavum and 9 from the rectum (Table S4). Of the 36 infants, 13 infants died within the first month after birth; of which, 12 in the group of infected infants and 1 in the group of colonized infants. When death occurred, it occurred on a median of 8 days (IQR 4–11) after birth. The lipid A of 7 of the 18 blood culture isolates contained 2OH-C14 (Table S1) (*E. bugandensis* n = 5 and *E. hormaechei* n = 2). In LPS H9, 2-hydroxymyristate (2OH-C14) was undetectable in the MALDI spectrum (absence of peaks at *m/z* 1813.4 and 1841.4; Table 2) and almost absent by GC-MS analysis (0.5%) as compared to the mean amount of this constituent in other strains (4.3 ± 1.4%). Therefore, we considered that LPS H9 is devoided of 2OH-C14.

**Figure 9:**
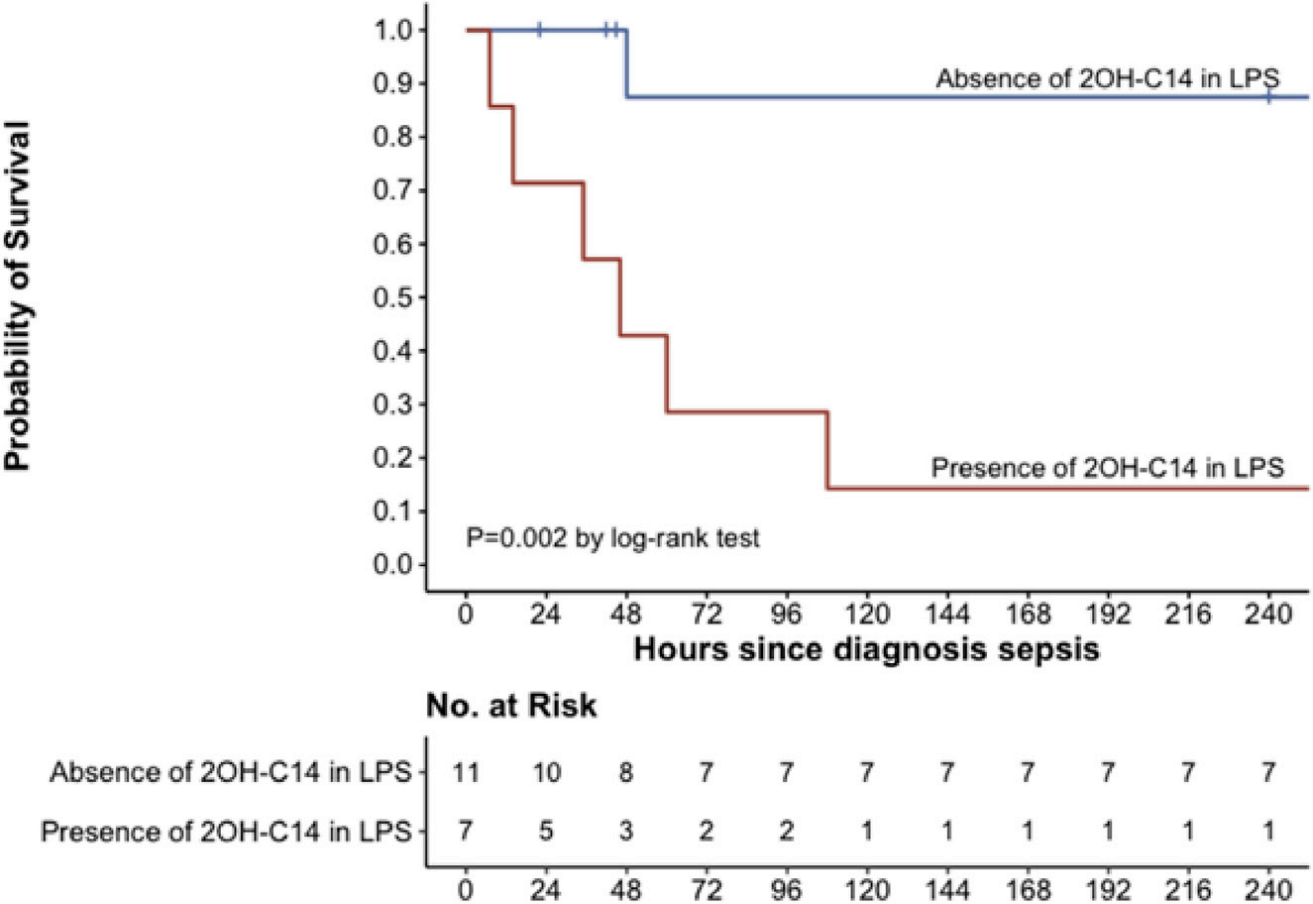
Kaplan–Meier survival curves in bloodstream infections due to *E. cloacae* complex, as a function of the presence or absence of 2OH-C14 on LPS Lipid A Red line: presence of 2OH-C14 on LPS Lipid A; Blue line: absence of 2OH-C14 on LPS Lipid A. Log rank test was performed using the R Statistical Software. p value < 0.05 was considered as significant.

Patients with *E. cloacae complex* septic shock and 2OH-C14 on LPS Lipid A were compared with those without the marker. Patients with LPS bearing 2OH-C14 had higher VasoInotropic score (p = 0.03) and were more likely to develop oliguria (p = 0.02) than those without 2OH-C14. Excluding deaths nonattributable to *E. cloacae* complex sepsis, the hazard ratio for death in children with *E. cloacae* complex septic shock group with 2OH-C14, as compared with the group without 2OH-C14, was 13.5 (95% CI, 1.61 to 113.1; *p = 0.016*). Survival curve of infants with septic shock with or without the presence of 2OH-C14 showed a significant difference and confirm the pathophysiologic role of 2OH-C14 in *E. cloacae* complex neonatal sepsis (Figure 9). Six deceased infants without presence of 2OH-C14 (infants H6, H8, H23, H24, H27, H28) died of pulmonary hemorrhage, meningitis, necrotizing enterocolitis, or cerebral hemorrhage, conditions not related to the *E. cloacae complex* septic shock.

## Discussion

Here, we provide the first evidence that presence of 2-hydroxymyristic acid moiety on lipid A structure is directly associated with mortality in neonates with *E. cloacae* complex septic shock, linking endotoxins structures with sepsis outcome.

Bacteria employ several means to protect themselves from adverse environmental stimuli, including exposure to antibiotics. Most of these strategies target the cell surface and are related in part to LPS changes, including modification of their O antigen or of their lipid A moieties [Simpson and Trent, 2019]. One strategy used by bacteria to protect themselves includes deacylation, hydroxylation, and palmitoylation by PagP of their lipid A (the biologically active region). With regard to deacylation, the retention of a primary 3-hydroxytetradecanoic acid at the 3-O position in the lipid A of *E. cloacae* complex is presumably due to the lack of expression of the PagL deacylase, which has been associated in *P. aeruginosa* with enhanced resistance to β-lactam antibiotics [Ernst et al., 2006]. Concerning palmitoylation, in the *E. cloacae* complex, species described here a secondary C16:0 substituent can be fixed either on position 2 (Figure 7B) or on position 3' (Figure 7A). This reminds the result obtained with *Bordetella parapertussis* lipid A where a secondary C16:0 substituent is present at both positions 2' and 3' [Petrou et al., 2016]. However, in the *E. cloacae* complex, these two positions are not simultaneously substituted with C16:0 (absence of a molecular species containing two C16:0 residues).

Other alterations of the lipid A moiety can be achieved through the addition of phosphoethanolamine and 4-amino-4-deoxy-L-arabinose (L-Ara4N), which decreases the net negative charge of lipid A. Interestingly, one of these frequent cationic binding group (e.g., ethanolamine) has not been found in lipid A from the *E. cloacae* complex, thus pointing to a preferential role for L-Ara4N.Unlike in *E. coli* and *S. enterica* where L-Ara4N is mainly fixed at the P4′ site by the relevant glycosyltransferase ArnT [Trent et al., 2001], we found that this phosphate substituent is linked to position 1 in the *E. cloacae* complex. This is due to the fact that the site of transfer of L-Ara4N depends on the structural features of the homolog of ArnT present in a given bacteria. For example, ArnT from *Cupriavidus metallidurans* yields only a single lipid A species modified at the 1-phosphate position, whereas ArnT from *S. enterica* serovar Typhimurium adds L-Ara4N to both the 1 and 4′ phosphates of lipid A [Petrou et al., 2016]. In particular, in lipid A precursors that accumulate in *S. typhimurium* mutants defective in Kdo biosynthesis, L-Ara4N is linked exclusively to the 1-phosphate [Zhou et al., 2000]. In another bacterium belonging to the Enterobacteriaceae family, *Yersinia pestis*, the substituent L-Ara4N is also in both the 1' and 4′ positions [Knirel et al., 2005]. Substitution of position 1 by L-Ara4N has also been found outside Enterobacteriaceae: Ernst et al. reported that L-Ara4N can also be attached to the 1-phosphate in a penta-acylated form of *P. aeruginosa* lipid A isolated from cystic fibrosis patients [Ernst et al., 2007]. The lipid A of *Burkholderia cenocepacia* contains also an L-Ara4N substituent on position 1 [De Soyza et al., 2008].

It has been noted by Zhou et al. [Zhou et al., 2001] that in a *Salmonella* mutant lacking L-Ara4N, a lipid A species containing a 1-pyrophosphate moiety was detectable. On the other hand, *E. coli* induced to incorporate L-Ara4N by culture in vanadate was no longer able to produce a 1-pyrophosphate variant of lipid A. To explain these puzzling observations, the authors suggested that incorporation of an L-Ara4N group is incompatible with the biosynthesis of the lipid A 1-pyrophosphate, possibly because of competition for a common donor substrate. Our results show that this is not the case in *E. cloacae* complex since many strains of this microorganism can produce the two types of lipid A: with a 1-pyrophosphate or a 1-aminoarabinose moiety.

One of the main objectives of our study was to identify relationships between the structures of the major biologically active component of the bacteria – lipid A – and the proinflammatory effect of the bacterial portage. The difficulty was that each *E. cloacae* complex strain bears multiple forms of LPSs that differ even in their lipid A regions. However, a mathematical analysis of the correspondence between the presence of the different molecular species and the IL-6 response of THP-1 cells allowed us to determine a coefficient of contribution of each species to the overall inflammatory response (Table 3). Four major contributors emerged (molecular species at *m/z* 1717.4, 1388.7, 1928.5, and 1956.6), with contribution factors higher than 70. The species at *m/z* 1717.4 results from the elimination of a phosphate group from the species at *m/z* 1797.4 by the action of a lipid A phosphatase. Cleavage by a 4′-phosphatase is unlikely because this type of phosphatase would also act on the species at *m/z* 1928.5 and lead to a peak at 1748.5, which is not observed. Therefore, the species at *m/z* 1717.4 is likely due to a 1-phosphatase as already found in *Francisella* and *Rhizobium* [Wang et al., 2006; Karbarz et al., 2003]. The second important contributor to inflammation is the species at *m/z* 1388.7. The production of this tetra-acylated species is likely due to the loss, by enzymatic cleavage, of a myristoxy-myristoyl residue from the hexa-acylated form of lipid A at *m/z* 1825.4. This cleavage is typically due to a homolog of LpxR, a Ca2+-dependent outer membrane lipase which cleaves the complete 3′-acyloxyacyl moiety of lipid A [Reynolds et al., 2006]. The fact that the peak at *m/z* 1388.7 is present in only five of the twelve strains studied indicates that the activity of this homolog of LpxR in the *E. cloacae c*omplex must be regulated. The mechanism of this regulation within the outer membrane is unknown but would certainly deserve particular attention because it can be expected that removal of the acyloxyacyl chain would substantially influence the recognition of LPSs by its TLR4:MD2 receptor complex.

It is generally accepted that for complete activation of the TLR4:MD-2 complex, a lipid A structure with six acyl chains and two phosphate groups is critical [Park et al., 2009]. Here, we see that the "incomplete" *E. cloacae* complex molecular species, at *m/z* 1388.7 and 1717.4, which do not meet those conditions, are nevertheless the best contributors to proinflammatory activity. Such theoretic discordance is also illustrated in *Neisseria meningitidis* expressing PagL deacylase that provides tetra-acylated LPS species but is biologically more active than the LPS from wild-strain *Neisseria meningitidis* [Zariri et al., 2016]. This paradoxical observation may result from the fact that the LPS:TLR4:MD2 complex triggers signaling via both the MyD88 and TRIF pathways as well as LPS recognition may be modulated by others proteins and cofactors. It is therefore possible that in conditions as encountered during sepsis where several molecular species of lipid A are present, the joint existence of the two TLR4:MD2 signaling pathways may allow incomplete forms of lipid A to contribute to inflammation through synergistic or antagonistic effects. The only two species containing L-Ara4N (at *m/z* 1928.5 and 1956.6) are also important contributors to inflammation. However, they are present in eight out of twelve LPSs (Table 2) and therefore cannot explain the differences in their inflammatory activity alone.

Pathogenicity of *E. cloacae* complex sepsis is multifactorial, with the involvement of a number of putative virulence factors. Barnes et al. [Barnes et al., 1997] reported that *E. cloacae* complex strains produce enterotoxins, α-hemolysin, and pore-forming cytotoxins after adhesion to epithelial cells. In addition, Krzyminska et al. [Krzyminska et al., 2009, 2010] found that genes of the type III secretion system that delivers bacterial toxins directly into the host cells are present in 27% of clinical isolates of *E. cloacae* complex, indicating that these bacteria may destroy phagocytes and epithelial cells and then disseminate with the host interstitial tissue. It has also been demonstrated that *E. cloacae* complex strains may induce apoptosis of human epithelial intestinal cells [Raetz, 2001]. This may represent another strategy of the *E. cloacae* complex, leading to tissue destruction and bacterial spreading resulting in systemic infection. Endotoxins remain a major, and well recognized, pathogenic effector of septic shock. In our patients, the fulminant and lethal course of the disease was suggestive of acute endotoxinemia. Whether patients with asymptomatic *E. cloacae* complex carriage had different endotoxins structures than patients with septic shock was obvious. Identification of small amounts of α-hydroxymyristic acid (2OH-C14) replacing the secondary C14:0 chain in position 2 (Figure 7A) or in position 3' (Figure 7B) in patients with septic shock and not in patients with asymptomatic carriage urges to re-evaluate its role in Enterobacteriaceae pathogenesis and 2OH-C14 biosynthesis. In many bacteria (*Salmonella, K. pneumoniae, P. aeruginosa, Bordetella pertussis, Legionella pneumophila*) the *lpx*O gene encodes a dioxygenase that transforms into 2OH-C14 a secondary myristate exclusively the 3′ position [Raetz, 2001; Llobet et al., 2015; Bartholomew et al., 2019; Lo Sciuto et al., 2019]. In other bacteria such as several *Bordetella* species (*Bordetella avium, Bordetella trematum, Bordetella hinzii,* and *Bordetella petrii*) a homolog of lpxO acts on the secondary substituted chains in positions 2 and 2' (and not in 3′ as in *Salmonella*) [Novikov et al., 2014].

Our results showing that 2OH-C14 is a secondary substituent at position 2 in some molecular species (Figure 7A) and 3′ in others (Figure 7B) suggest that homologs of these two variants of LpxO are both present in the *E. cloacae* complex*.* It should be noted that 2OH-C14 may be absent, even in the presence of LpxO, if an enzyme homologous to LpxL2 is absent. Indeed, LpxO only modifies a secondary myristate group previously transferred by LpxL2. Only one study reported the virulence of *E. bugandensis* in the NICU (strain EB-247) [Pati et al., 2018]. *In silico* analysis of EB-247 genome shows the absence of *lpxO* homolog (GenBank: NZ_LT992502). After isolating lipid A moieties from strain EB-247, no peak corresponding to the 2-hydroxymyristate residue (*m/z* 1813.4 and 1841.4) were identified from the MALDI-TOF spectrum (Figure S2) and LPSs analysis by GC-MS did not retrieve α-hydroxymyristic acid (data not shown).

Our analyses of lipid A structures by MALDI-TOF, followed by GC-MS analyses of their fatty acid content, showed for the first time an indisputable correlation between the presence of 2-hydroxymyristic acid on lipid A and mortality due to *E. cloacae complex* septic shock (Figure 8). In contrast, lethality was neither correlated with any other structural features nor species identification within the *E. cloacae* complex and phenotypic markers such as ERIC-PCR profiles or overproduction of AmpC cephalosporinase. The role of 2-hydroxymyristate as a virulence factor is also supported by Merritt et al. with *B. pseudomallei* [Merritt et al., 2006]. The effect of 2-hydroxymyristate could be to increase hydrogen bonds between adjacent lipid A chains, which would increase the membrane's resistance to the penetration of antimicrobial cationic peptides used as defense mechanisms. Raetz's group [Gibbons et al., 2000] proposed another interesting hypothesis that could explain the role of 2OH-C14 as a virulence factor: during infection, 2-hydroxymyristic acids released by LPS through leukocyte acyloxyacyl hydrolase would be converted to 2-hydroxymyristoyl coenzyme A, a well-known potent inhibitor of N-myristoyl transferase that is required for cell signaling. This would gradually destroy, by apoptosis, the immune cellular effectors.

Identification of 2-hydroxymyristic acid as a virulence factor and prognosis markers in *E. cloacae* complex sepsis opens the possibility of using it as a marker of clinical severity, marker of antibiotics therapy initiation, as well as *E. cloacae* complex pathogenicity stratification.

### Limitations of study

Our findings need to be confirmed in larger studies and expended to other gram-negative bacteria such *K. pneumonia* and other *Enterobacter* encountered in patients with septic shock.

## STAR★Methods

### Key resources table

**Table:** 

REAGENT or RESOURCE	SOURCE	IDENTIFIER
**Deposited data**		

Repository data from this study	Anonymous patient data upon request	NA

**Software and algorythms**		

R Studio (R core Team, 2018; R Foundation for statistical Computing)	https://www.r-project.org/	NA

### Ressource availability

#### Lead contact

Further information and request for resources and reagents should be directed to and will be fulfilled by the lead contact, Pierre Tissières (pierre.tissieres@i2bc.paris-saclay.fr).

#### Materials availability

This study did not generated new unique reagents.

#### Data and code availability

Data reported in this paper will be shared by the lead contact upon request. This paper does not report original code. Any additional information and requests for ressources and reagents should be directed and will be fullfield by the lead contact.

### Experimental model and subject details

#### Patients

Retrospective analysis of all newborn hospitalized in the NICU of the Béclère Hospital with *E. cloacae* complex septic shock. Septic shock was defined as a sepsis associated with more than two organ failure requiring vasoinotropic support. The study was approved by ethical committee of the French Society of Intensive Care (CE SRLF 19–40). Legal representatives were informed and agreed on the use of the clinical data. Gender of patients was equally balanced (male n = 15; female n = 21). All patients were neonates with a median gestational age of 27 weeks (See Table S1). Influence of gender, age and developmental stage may have influence host response to *E. cloacae* infection and were not studied.

### Method details

#### Identification, antimicrobial susceptibility testing, molecular typing of *E. cloacae complex*

*E. cloacae* complex isolates from blood cultures or colonization sites (cavum) in hospitalized infants were obtained from the microbiology laboratory of Antoine Beclere hospital. Bacteria were inoculated on Columbia agar with 5% sheep blood ((bioMérieux, Marcy l’Étoile, France) and were incubated overnight at 37°C. Identification of the *E. cloacae* complex was performed with mass spectrometry (MALDI-TOF-MS) (Brucker, Leipzig, Germany) according to the manufacturer's instructions. In addition, we distinguished the different species among the *E.cloacae* complex as previously described [Hoffmann and Roggenkamp,2003]. Antimicrobial susceptibility testing was performed using agar disk diffusion method on Mueller-Hinton (MH, Bio-Rad, Hercules, CA) according to the Eucast guidelines (http://www.eucast.org/). Enterobacterial repetitive intergenic consensus PCR (ERIC-PCR) was performed according to Duan [Duan et al., 2009]. ERIC banding patterns obtained from the agarose gel electrophoresis were used to define different profiles.

#### LPS and lipid A extraction and analysis

We selected twelve of *E. cloacae* complex strains from blood cultures (n = 8) and from colonization sites (n = 4) which differed in several of their characteristics, including ERIC-PCR profiles, antibiotic resistance profiles or identification species to lipid A extraction and analysis (Table S2). All MALDI-TOF and GC-MS experiment were representative of two to five independent experiment in triplicate.

Bacteria were grown overnight at 37°C in LB broth (Sigma Aldrich, St. Louis, Missouri, MI), and LPSs were isolated by the phenol/water method of Westphal and Jann [Westphal and Jann, 1965]. Briefly, the wet pellet of bacteria was stirred in 45% aqueous phenol at 65°C for 30 min, insoluble material was removed from the cooled water phase by centrifugation and the clear extract was dialyzed under running tap water until free of phenol, then dialyzed against distilled water [Chafchaouni-Moussaoui et al., 2011]. Extracts were subjected to enzyme treatments (DNAse, RNAse, and proteinase K) to remove DNA, RNA, and proteins, and further purified with acidified chloroform-methanol-water to remove contaminating phospholipids and lipoproteins. LPSs were then washed by suspension in cold methanol, centrifuged (7000 × g) and dried under a stream of air. Lipid A was prepared by the triethylamine-citrate method [Chafchaouni-Moussaoui et al., 2011]. Briefly, the LPS sample was suspended at a concentration of 10 μg/μL in a 0.01 M triethylamine-citrate solution (1:1 molar ratio, pH 3.6) and heated for 1 hr at 100°C. The sample was then lyophilized and suspended in methanol. After centrifugation (7000 × g for 10 min at 4°C), lipid A was extracted with a mixture of chloroform: methanol: water (3: 1.5: 0.25; by vol.) at a concentration of 10 μg/μL. The molecular species present in this preparation were analyzed using a AXIMA performance (Shimadzu Corp., Kyoto, Japan) matrix-assisted laser desorption ionization–time of flight (MALDI-TOF) mass spectrometer (Institute of Integrative Biology of the Cell, CNRS, CEA, Paris Saclay University, Gif-sur-Yvette, France). A suspension of lipid A (1 μg/μL) in chloroform: methanol: water (3:1.5:0.25, v:v:v) was desalted with a few grains of Dowex 50W-X8 (H+), 1 μL was deposited on the target mixed with 1 μL of a gentisic acid (2,5-dihydroxybenzoic acid) matrix (Sigma Aldrich, St. Louis, Missouri, MI) suspended at 10 μg/μL in the same solvent or in 0.1 M aqueous citric acid, and dried. Analyte ions were desorbed from the matrix with pulses from a 337 nm nitrogen laser. Spectra were obtained in the negative-ion mode at 20 kV, with the linear detector. Mass calibration was performed with the peptide mass standards kit of AB SCIEX, or with a purified and structurally characterized LPS sample from *B. pertussis* and *E. coli J5* prepared in our laboratory.

#### Fatty acid composition of LPSs analyzed by gas chromatography/mass spectrometry

The LPS samples (200 μg) were incubated at 85°C for 15 to 18 hr in 600 μL of a mixture of anhydrous methanol/acetyl chloride (10/1.5 by vol.) containing 4 μg of arachidic (eicosanoic) acid (C20) used as an internal standard. After this transmethylation reaction, methanol was evaporated under vacuum at room temperature, the resulting fatty acid methyl esters were extracted in ethyl acetate (600 μL), and the solvant was evaporated at room temperature under a stream of nitrogen. The material was dissolved in 50 μL of ethyl acetate and the solution (1–5 μL) was analyzed by Gas Chromatography coupled to Mass Spectrometry (GC-MS) in a Shimadzu (GCMS-QP2010SE) apparatus. A capillary column from Phenomenex (Zebron ZB-5MS, 30 m × 0.25 mm × 0.25 μm) was used with a temperature gradient from 50°C to 120°C (20°C/min) followed by a gradient from 120°C to 250°C (3°C/min) and finally a constant temperature (250°C for 2 min). Fatty acids were identified by their mass spectra and their retention times compared to fatty acid standards (Sigma Aldrich, St. Louis, Missouri, MI).

#### Endotoxins cytokines assay

THP-1 cell (ATCC, Old Town Manassas, VA) were cultured at 37°C in humid air with 5% CO2, in RPMI 1640 medium supplemented with 10% heat-inactivated (56°C, 30 min) fetal bovine serum (Gibco, ThermoFisher Scientific, Waltham, MA), 100 IU/mL penicillin and 100 μg/mL streptomycin (Sigma Aldrich, St. Louis, Missouri, MI). Viable THP-1 cells (10^6^ cells in 2 mL of culture medium) were added to each well of tissue culture-treated, flat-bottomed, non-pyrogenic, polystyrene 6-well plates and stimulated with 1 ng/mL LPS, no LPS added was the control. The LPS extracted from *E. coli* J5 (ATCC, Old Town Manassas, VA) and purified as indicated above, was used as a reference. *In vitro* stimulations were performed in triplicate for 24 hr and supernatants were stored at −20°C for ELISA analysis. In the cell-free supernatants, transferred into microplates (96-well Maxisorp Nunc), TNF-α and IL-6 levels were determined by ELISA kits (Ready Set Go, e-Bioscience, ThermoFisher Scientific, Waltham, MA). In each well, the OD at 450 nm was measured with a microplate reader (Multiskan EX, ThermoFisher Scientific, Waltham, MA).

#### Lipid A structures pro-inflammatory potency

To calculate specific contributions of each lipid A molecular species, mathematic model was constructed. LPSs from twelve strains and eighteen molecular species (MALDI peaks), provide twelve equations and eighteen unknowns. But we can limit the number of unknowns by making three reasonable approximations: i) The species 1599.1 and 1783.4 have a contribution coefficient of 0 (little influence) because present in only one strain. ii) The species 1813.4 and 1841.4 have the same coefficient (only 2 strains differ by them) and iii) The species 1928.5 and 1956.5 have also the same coefficient, for the same reason. This leaves a system of twelve equations with only twelve unknowns, where each equation indicates the number of times (0,1,2, or 3) that a molecular species contributes to the inflammatory activity of a given LPS. These presences can be represented by a square matrix. To calculate each structure contribution, it is then sufficient to solve the matrix equation: (Matrix of presence) x (Vector of contributions) = IL-6 concentration (pg/mL). Many computer applications designed to solve linear systems can handle this matrix equation. The result is that this linear system admits only one solution.

#### Screening of *lpxO* by real-time PCR

Isolates were screened for the presence of *lpxO* with a real-time PCR with primers (*lpxO*-F 5′-GGCTCGGTTCGTTACCATCT-3′; *lpxO*-R 5′-AATAAAACGGATCAAACGCG-3′). Bacterial DNA was isolated with easyMag (bioMérieux, Marcy-l'Étoile, France). Realtime *lpxO* PCR was performed using the following PCR mix conditions: 12.5 μL of Master mix GoTtaq qPcr Promega (Promega, Madison, WI), 9 μL sterile water and 0.2 μM of each primer in a total volume of 25 μL (including 2.5 μL of template). PCR protocol was performed under the following conditions: pre-PCR of 10 min at 95°C to fully denature the template DNA and activate the polymerase followed by 40 cycles of 15 s at 94°C for denaturation and 30 s at 60°C for annealing. Amplification and detection were carried out in an Applied Biosystems Prism 7500 System (ThermoFisher Scientific, Waltham, MA).

### Quantification and statistical analysis

#### Statistical analysis

Kaplan-Meier survival curves were drawn and Log rank test was performed using the R Statistical Software. p value < 0.05 was considered as significant.
