# Supplementary material for: Presence of 2-hydroxymyristate on endotoxins is associated with death in neonates with Enterobacter cloacae complex septic shock
Source: iScience. 2021 Jul 30;24(8):102916. doi: 10.1016/j.isci.2021.102916 (PMC8361193; doi:10.1016/j.isci.2021.102916)
Supplement: Document S1. Figures S1 and S2 and Tables S1–S4 [file mmc1.pdf]

iScience, Volume *24*

Supplemental information

Presence of 2-hydroxymyristate on endotoxins is associated with death in neonates with *Enterobacter cloacae* complex septic shock

Luis A. Augusto, Nadège Bourgeois-Nicolaos, Aude Breton, Simon Barreault, Enrique Hernandez Alonso, Stuti Gera, Véronique Faraut-Derouin, Nada Semaan, Daniele De Luca, Richard Chaby, Florence Doucet-Populaire, and Pierre Tissières

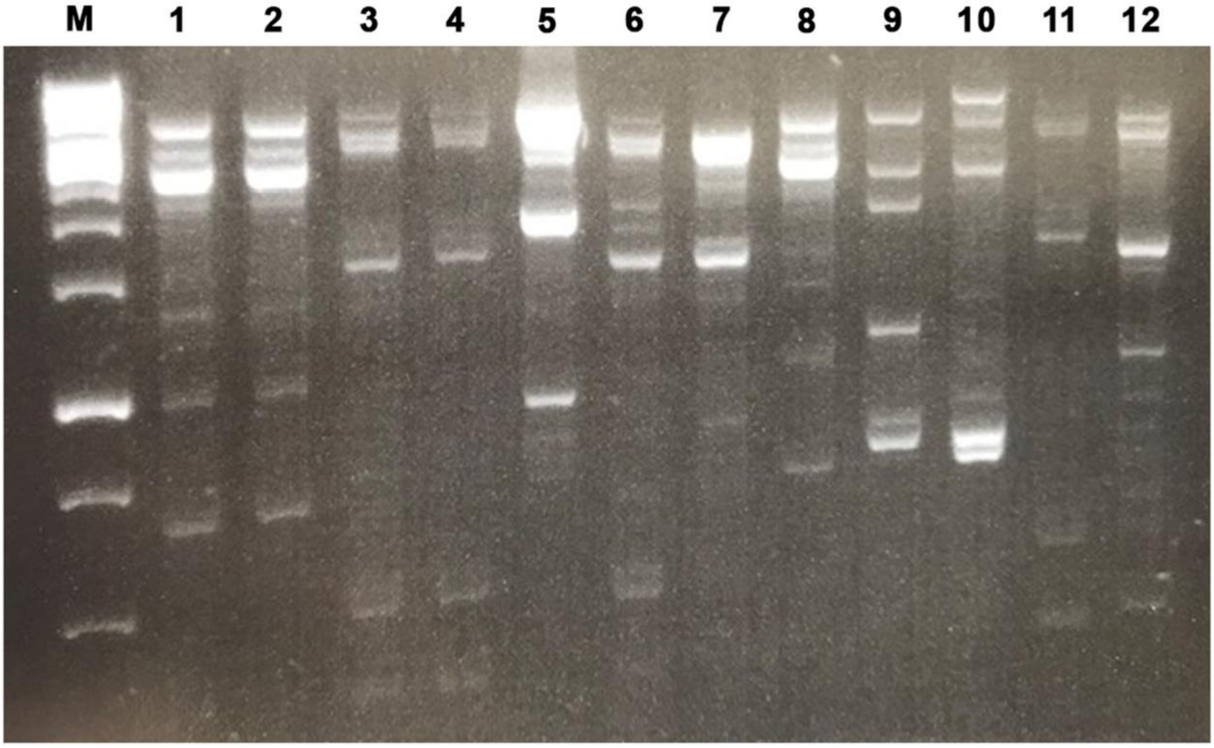

Figure S1 : Nine different enterobacterial repetitive intergenic concensus (ERIC) – polymerase chain reaction (PCR) profiles were identified, related to STAR methods. ERIC-PCR banding patterns of 12 *E. cloacae complex* isolates analysed on a 1.5 % agarose gel. Slots from left to right: M, 100bp DNA size Marker; 1, H1 profile A; 2, H2 profile A; 3, H7o profile E; 4, H7i profile E; 5, H8 profile C; 6, H9 profile B; 7, H10 profile F; 8, H11 profile A; 9, C12 profile H; 10, C16 profile G; 11, C17 profile E; 12, C18 profile F.

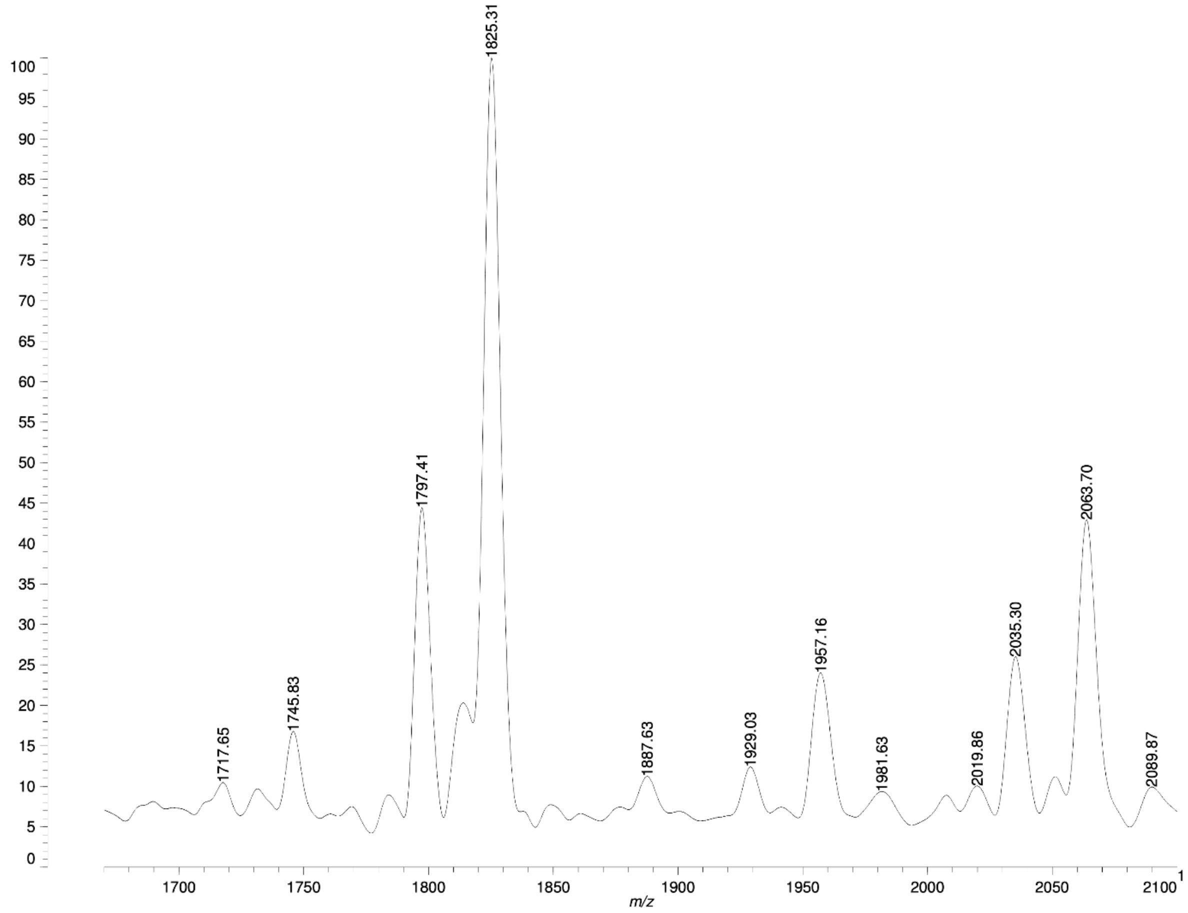

Figure S2: No peak corresponding to the 2-hydroxymyristate residue (*m/z* 1813.4 and 1841.4) were identified from the MALDI-TOF spectrum of isolated lipid A moieties from strain EB-247, related to STAR methods.

Table S1. Patients characteristics, related to STAR methods

	Overall (n = 36)	ECC sepsis (n = 18)	ECC colonization (n = 18)	*p value*†
**Gestational age (weeks)**	27.0 (25.8 ; 28.8)	27.3 (25.0 ; 28.8)	26.9 (26.3 ; 28.6)	*0.74*
**CRIB II score**	10 (8 ; 12)	10 (8 ; 13)	10 (8 ; 12)	*0.75*
**Male gender**	15 (42%)	8 (44%)	7 (39%)	*1.00*
**Weight (grams)**	917 (728 ; 1085)	838 (585 ; 1080)	927 (800 ; 1080)	*0.32*
**Clinical chorioamnionitis**	13 (36%)	5 (28%)	8 (44%)	*0.49*
**Antibiotics within 72 h of birth**	13 (36%)	4 (22%)	9 (50%)	*0.16*
**Maximum VIS score #**	0 (0 ; 63)	75 (0 ; 200)	0 (0 ; 0)	<*.001*
**Death**	14 (39%)	12 (67%)	2 (11%)	*0.001*
**Age at death (days)**	8 (4 ; 11)	7 (4 ; 10)	52 (31 ; 73)	*0.14*

CRIB : clinical risk index for babies. VIS : vasoactive-inotropic score.
* Data are reported as numbers (with percentages) or medians (with interquartile ranges). † Wilcoxon-Mann-Whitney or Fisher's exact test, as appropriate. α = 0.05.
# VIS score = 100 x (epinephrine + norepinephrine in mcg/kg/min) + (dobutamine + dopamine in mcg/kg/min) + 10,000 x (vasopressin in U/kg/min) + 10 x (milrinone in mcg/kg/min).

Table S2. Characterization of *E. cloacae complex* strains from blood culture or cavum swab in the NICU setting, related to STAR methods.

Souche	source of isolation	Espèces	ERIC-PCR profile	Overproduction of ampC	marqueur	death
H1*	blood	*E. hormaechei*	A	Y	6.3	+
H2*	blood	*E. hormaechei*	A	Y	0	-
H3	blood	*E. hormaechei*	A	Y	2.6	+
H4	blood	*E. bugandensis*	E	N	5.4	+
H5	blood	*E. bugandensis*	E	N	4.2	+
H6	blood	*E. hormaechei*	A	Y	0	+
H7o*	blood	*E. bugandensis*	E	Y		+
H7i*	blood	*E. bugandensis*	E	N	4.9	+
H8*	blood	*E. hormaechei*	C	N	0	+
H9*	blood	*E. bugandensis*	B	N	0.5	-
H10*	blood	*E. bugandensis*	F	N	4.5	+
H11*	blood	*E. hormaechei*	A	Y	0	-
H21	blood	*E. hormaechei*	I	N	0	-
H23	blood	*E. hormaechei*	A	N	0	+
H24	blood	*E. hormaechei*	A	Y	0	+
H25	blood	*E. hormaechei*	J	N	0	-
H26	blood	*E. cloacae*	G	Y	2.2	-
H27	blood	*E. bugandensis*	F	N	0	+
H28	blood	*E. hormaechei*	H	N	0	+
C12	cavum swab	NP	H	N	NP	-
C16	cavum swab	NP	G	N	NP	-
C17	cavum swab	NP	E	N	NP	-
C18	cavum swab	NP	F	N	NP	-

Y: Yes ; N: No; NP, not performed; * strains selected to lipid A extraction and analysis

Table S3 : Clustering of the twelve *E. cloacae* strains according to the presence or absence of four substituents in their lipid A, related to STAR methods

	L-Ara4N^{+} / PP^{+}	L-Ara4N^{+} / PP^{-}	L-Ara4N^{-} / PP^{+}
2OH-C14^{+} / C13^{-}	H1 (✝), H7h (✝), H7i (✝), H10 (✝)		
2OH-C14^{-} / C13^{+}	H2, H8 (✝)		C12
2OH-C14^{-} / C13^{-}	H9, C18	H11	C17, C16

✝ : deceased infant ; L-Ara4N : 4-amino-4-deoxy-L-arabinose ; PP : pyrophosphate ; 2OH-C14 : 2-hydroxymyristic acid ; C13 : tridecanoic acid. "+" indicates the presence and "-" the absence of the corresponding substituent.

Table S4 : Neonatal lethality of thirty-six strains of E. cloacae complex in relation to 2OH-C14, related to STAR methods.

Infant statut	Strain			2OH-C14 (%)**	*LpxO* gene ***	Infant death ****
	source of isolation	Designation	AmpC expression*			
colonised	Cavum	C1	i	6.3	+	+
		C2	i	0	-	-
		C3	i	0	-	-
		C4	i	0	-	-
		C6	i	0	-	-
		C7	i	0	-	-
		C9	i	0	-	-
		C21	i	0	-	-
		C25	i	0	-	-
	Rectum	C5	o	0	-	-
		C8	o	0	-	-
		C10	o	0	-	-
		C11	o	0	-	-
		C23	o	4.7	+	-
		C24	o	4.9	+	-
		C26	o	0	-	-
		C27	i	0	-	-
		C28	i	0	-	-
infected	Blood	H1	o	6.3	-	+
		H2	o	0	-	-
		H3	o	2.6	-	+
		H4	o	5.4	+	+
		H5	i	4.2	+	+
		H6	o	0	-	+
		H7	o	4.9	+	+
		H8	i	0	-	+
		H9	i	0.5	-	-
		H10	i	4.5	+	+
		H11	o	0	-	-
		H21	i	0	-	-
		H23	i	0	-	+

		H24	o	0	-	+
		H25	i	0	-	-
		H26	o	2.2	+	-
		H27	i	0	-	+
		H28	i	0	-	+

* inducible (i) or overproduced (o) cephalosporinase.
** expressed as the ratio (%): [2OH-C14] / [3OH-C14] x 100.
*** +: detected ; - not detected
**** +: deceased infant within the first month after birth; - survived infant
